# Biodiversity and Classification of Phages Infecting *Lactobacillus brevis*

**DOI:** 10.3389/fmicb.2019.02396

**Published:** 2019-10-16

**Authors:** Marine Feyereisen, Jennifer Mahony, Horst Neve, Charles M. A. P. Franz, Jean-Paul Noben, Tadhg O’Sullivan, Viktor Boer, Douwe van Sinderen

**Affiliations:** ^1^School of Microbiology, University College Cork, Cork, Ireland; ^2^APC Microbiome Ireland, University College Cork, Cork, Ireland; ^3^Department Microbiology and Biotechnology, Federal Research Centre of Nutrition and Food, Max Rubner-Institut, Kiel, Germany; ^4^Department Physiology Biochemistry and Immunology, Biomedical Research Institute, Hasselt University, Diepenbeek, Belgium; ^5^HEINEKEN Global Innovation and Research, Heineken Supply Chain B.V, Zoeterwoude, Netherlands

**Keywords:** lactic acid bacteria, beer spoilage, bacteriophage, lysogeny, prophage, genomic analysis, induction, *Myoviridae*

## Abstract

*Lactobacillus brevis* is a lactic acid bacterium that is known as a food and beverage spoilage organism, and more specifically as a beer-spoiler. Phages of *L. brevis* have been described, but very limited data is available regarding temperate phages of *L. brevis*. Temperate phages may exert benefits to the host, while they may also be employed to combat beer spoilage. The current study reports on the incidence of prophage sequences present in nineteen distinct *L. brevis* genomes. Prophage induction was evaluated using mitomycin C exposure followed by genome targeted-PCR, electron microscopy and structural proteome analysis. The morphological and genome sequence analyses revealed significant diversity among *L. brevis* prophages, which appear to be dominated by members of the *Myoviridae* phage family. Based on this analysis, we propose a classification of *L. brevis* phages into five groups.

## Introduction

The most prevalent spoilage bacteria associated with beer fermentations are members of the lactic acid bacteria (LAB), which account for approximately 70% of all microbial spoilage incidents (Vaughan et al., 2005; Bokulich and Bamforth, 2013; Garofalo et al., 2015). *Lactobacillus brevis* strains are frequently reported to be the cause of such spoilage events as they have developed mechanisms to survive and grow in beer (Suzuki et al., 2006). Strains of this species can be found on raw materials used in breweries and represent a major microbial contaminant during the production and storage of beer. Bacterial strains have acquired features throughout evolution allowing them to become more robust including resistance to virulent bacteriophages (Forde and Fitzgerald, 1999; Brüssow, 2001; Mahony et al., 2016b). Moreover, the chromosomes of the majority of LAB are known to harbor one or more prophage regions and their presence may benefit the host by providing resistance attributes to its environment (Canchaya et al., 2003). Upon induction, these phages enter the lytic cycle leading to bacterial cell death and formation of intact phage particles (Davidson et al., 1990), and therefore it is relevant to examine their presence and functionality (Kelleher et al., 2018). Prophages of LAB have been widely studied and particularly so in *Lactococcus lactis* (Kelleher et al., 2018) and *Streptococcus thermophilus* (Husson-Kao et al., 2000). Temperate phages of the genus *Lactobacillus* have been sequenced and are primarily classified as members of the *Siphoviridae* family (Villion and Moineau, 2009), being characterized by a non-contractile tail (Forsman, 1993). Successful induction of such prophages has been reported using UV, thermal exposure or treatment with DNA damaging/antimicrobial compounds such as mitomycin C and bacteriocins (Chopin et al., 1989, 2001; Meijer et al., 1998; Madera et al., 2009).

While the presence of prophages in LAB genomes is widely described, only a small number of studies have investigated their integrity and inducibility within these strains (Mittler, 1996; Lunde et al., 2003). In the case of beer spoilage by *L. brevis*, prophage induction represents a beneficial attribute that could be harnessed as a natural alternative to chemical compounds in the eradication of spoilage bacteria. Indeed, induction of prophages is expected to cause bacterial cell lysis thus avoiding the development of spoilage organisms in the brewing industry. Although prophages represent a reservoir for adaptation, prophages of *L. brevis* are currently poorly described. To date, the genome of a single temperate phage of *L. brevis*, namely LBR48, has been sequenced and characterized. The prophage was induced from *L. brevis* strain C30 using mitomycin C. LBR48 is 48 Kb long containing 90 putative open reading frames (ORFs) and was classified as a member of the *Myoviridae* family. Interestingly, the LBR48 genome does not show protein sequence similarity with any other *Lactobacillus* phages (Jang et al., 2011).

Advances in genome sequencing technologies have considerably increased the number of available bacterial genome sequences, improving also the detection capability of prophage-encoded regions within these genomes. In the current study, nineteen publically available *L. brevis* genome sequences were used to identify prophage-encoding regions. Five of these strains were available for prophage induction trials, allowing the assessment of the ability of these temperate phages to form intact phage particles and cause host cell lysis. The diversity of *L. brevis* temperate phages was studied to establish relatedness between *L. brevis* phages (temperate and virulent), resulting in a proposed classification scheme of *L. brevis* phages based on morphology and genome sequence data.

## Materials and Methods

### Bacterial Strains and Growth Conditions

*Lactobacillus brevis* strains used in this study are detailed in Table 1. Bacteria were cultured in MRS broth (Oxoid Ltd., Hampshire, United Kingdom) at 30°C.

**TABLE 1:** *Lactobacillus brevis* strains used in this study and prophage regions predicted by PHASTER.

**Strain name (isolation source)**	**Genbank accession**	**No. prophage regions detected by PHASTER**			**References**
		**Intact**	**Questionable**	**Incomplete**	
100D8^∗^ (Silage)	CP015338	1	1	1	
ATCC 367 (Silage)	CP000416	1	0	0	Makarova et al., 2006
BDGP6^∗^ (*Drosophila*’s gut)	CP024635	4	1	2	
KB290^∗^ (Fermented vegetable)	AP012167	2	0	1	Fukao et al., 2013
NPS-QW-145^∗^ (Kimchi)	CP015398	0	1	2	Wu et al., 2015
NCTC13768^∗^ (Unknown)	LS483405	0	1	0	
SA-C12 (Silage)	CP031185	2	1	1	Feyereisen et al., 2019a
SRCM101106^∗^ (Food)	CP021674	3	0	1	
SRCM101174^∗^ (Food)	CP021479	3	0	1	
TMW 1.2108^∗^ (Beer)	CP019734	2	0	0	Fraunhofer et al., 2018
TMW 1.2111^∗^ (Beer)	CP019743	2	0	0	Fraunhofer et al., 2018
TMW 1.2112^∗^ (Beer)	CP016797	1	0	0	Fraunhofer et al., 2018
TMW 1.2113^∗^ (Brewery surface)	CP019750	2	0	0	Fraunhofer et al., 2018
UCCLB521 (Brewery surface)	CP031208	0	0	2	Feyereisen et al., 2019a
UCCLB556 (Brewery surface)	CP031174	0	1	0	Feyereisen et al., 2019a
UCCLB95 (Beer)	CP031182	1	0	2	Feyereisen et al., 2019a
UCCLBBS124 (Beer)	CP031169	1	0	1	Feyereisen et al., 2019a
UCCLBBS449 (Beer)	CP031198	1	0	3	Feyereisen et al., 2019a
ZLB004^∗^ (Pig’s feces)	CP021456	1	0	0	

*^∗^These strains were not tested for prophage induction.*

### Prophage Identification and Genome Annotation

PHAge search tool enhanced release (PHASTER) (Zhou et al., 2011; Arndt et al., 2016) was used to screen for prophage-specifying DNA regions within the genome of available *L. brevis* strains. Intact prophages were manually annotated to confirm the presence of all expected genes required to produce a fully functional phage particle including genes encoding proteins associated with replication functions (e.g., replisome and DNA-binding proteins) packaging (small and large terminases), morphogenesis (e.g., capsid and tail) and lysis (holin and lysin). Genes required for lysogeny maintenance (e.g., integrase and repressor) were also investigated (Kelleher et al., 2018). Integration sites of prophages (attL and attR) were recorded and are presented in Supplementary Table S1. Prophage genome sequences were retrieved and annotated as previously described (Kelleher et al., 2018). Briefly, ORF prediction was performed using the Prodigal prediction software (Hyatt et al., 2010) and confirmed using BLASTX alignments (Altschul et al., 1990). The automatic annotations were refined using Artemis v16.0.0 to allow visual inspection of ORF predictions (Rutherford et al., 2000). Moreover, BLASTP (Altschul et al., 1997) and HHPred (Söding et al., 2005) analyses were performed to assign functional annotations to the predicted ORFs. Transfer RNA (tRNA) genes were predicted using tRNA-scan-SE v2.0 (Schattner et al., 2005) and added manually using Artemis.

### Phylogenetic Analysis

A proteomic tree was constructed using a concatenated amino acid sequence of all encoded proteins for each of the *L. brevis* phages sequenced to date. The concatenated amino acid sequence begins with the ORF encoding the small terminase subunit (TerS) (Casey et al., 2015). The concatenated sequences were aligned using ClustalW (Thompson et al., 2002). The phylogenetic tree was constructed using the neighbor-joining method and bootstrapped employing 1,000 replicates. The final tree was visualized using MEGA7 (Kumar et al., 2008).

### Genome Characterization and Organization

Following phylogenetic analysis, genomes of representative temperate phages were selected for further analysis where overall genome content and organization were studied. The genome content and architecture were analyzed based on the observation of the modular organization of the genomes into the following modules: packaging, morphogenesis, lysis, lysogeny, and replication. Protein sequences of representative phages were compared using all-against-all, bi-directional BLAST alignments (Altschul et al., 1990). An alignment cut-off *E*-value of 0.0001, and a similarity cut-off level of at least 30% amino acid identity across 80% of the sequence length was applied. This analysis allowed the amino acid similarity assignment between temperate phage genomes and the study of the overall genome similarity/diversity among *L. brevis* phages.

### Prophage Induction Trials

To assess the functionality of the identified prophage-encoding regions, prophage induction trials were performed using the DNA crosslinking agent mitomycin C (MitC). For this assay, five *L. brevis* strains were available for testing: ATCC367, SA-C12, UCCLB95, UCCLBBS124, and UCCLBBS449. 10 mL MRS broth was inoculated with 2% of a fresh overnight culture of the relevant bacterial strain. Cultures were incubated at 30°C until an OD_600__nm_ of 0.1, 0.2, or 0.3 was reached at which point 0.1, 0.2, or 0.3 μg/mL MitC (final concentration) was added. A high concentration of 2 μg/mL MitC (final concentration) was applied when cells reached an OD_600__nm_ of 0.2. This was performed to ascertain if cell lysis occurred due to prophage induction or lethal MitC toxicity. Indeed, MitC levels of between 0.1 and 0.3 μg/mL are relatively low and when induction occurs it would be considered genuine prophage-induction mediated cell lysis. Conversely, higher concentrations of MitC (e.g., 2 μg/mL MitC) are expected to cause growth arrest and cell death due to acute toxicity. Cultures were maintained at room temperature for 30 h during which the OD_600__nm_ was recorded at 60 min time intervals for the first 8 h and then at 15, 20, 25, and 30 h.

Using the same protocol as MitC prophage induction, potential induction regimes using other stress-inducing chemicals (2% v/v NaOH, 2% v/v formic acid or 2% v/v acetic acid) or physical treatment (direct UV light exposure (254 nm) of 30 min at a distance of 5 cm on a 1 cm height culture suspension) that resemble circumstances in the brewing industry for cleaning and sanitizing purposes were also assessed (Schmidt, 1997; Loeffler, 2006).

### Validation of Prophage Induction by DNA Sequencing and Electron Microscopy

To validate prophage induction from *L. brevis* strains cited above, the DNA derived from cell-free supernatants was extracted and sequenced. Phage DNA was isolated using a previously described phage DNA extraction protocol (Lavelle et al., 2018). Primers were designed based on prophage sequences in order to confirm the presence of induced prophages in the cell-free supernatants of MitC-treated cultures (Table 2).

**TABLE 2:** Primer sequences used to amplify specific regions of induced prophages.

**Primer name**	**Sequence (5′ – 3′)**	**Target**
TPMB095F	gaatcctggcgataactag	TMP region of TPMB095 prophage
TPMB095R	gtggcaccagcgtatcgaa	
TPMB449F	cttcaatcaccatctaag	TMP region of TPMB449 prophage
TPMB449R	gactatcagcaatcgcatt	
TPMB124F	ggttgccttctgcaagg	TMP region of TPMB124 prophage
TPMB124R	gttaaggaggtgtgactaa	
TPSAC12-1F	gtatggcaatcaagcacac	TMP region of TPSAC12-1 prophage
TPSAC12-1R	tgccatctcattggtgac	
TPSAC12-2F	gacttcataacagcaat	TMP region of TPSAC12-2 prophage
TPSAC12-2R	ggtccactaatggcgac	
TPATCC367F	ggaaccttgtcgttcata	TMP region of TPATCC367 prophage
TPATCC367R	gcagcttctctagcaccac	

To validate prophage induction using electron microscopy, MitC-treated cultures were harvested by centrifugation at 5000 × *g* for 10 min, after which the supernatant was filtered twice through a 0.45 μm filter prior to electron microscopy analysis. Transmission electron microscopy of the samples was performed as previously described (Casey et al., 2015). Negative staining was performed using 2% (w/v) uranyl acetate on freshly prepared ultrathin carbon films. Grids were analyzed in a Tecnai 10 transmission electron microscope (FEI Thermo Fisher Scientific, Eindhoven, Netherlands) at an acceleration voltage of 80 kV. Micrographs were taken with a MegaView G2 charge-coupled device camera (Emsis, Muenster, Germany).

### Phage Structural Proteome and Mass-Spectrometry

An aliquot (30 μL) of CsCl-purified phage sample was mixed with 10 μL of SDS loading buffer containing 50 mM β-mercaptoethanol. The structural protein profile was generated by standard Tris-glycine sodium dodecyl sulfate (SDS)–12% polyacrylamide gel electrophoresis (PAGE). Gel slices were then excised, trypsinized, and analyzed using electrospray ionization tandem mass spectrometry (ESI-MS/MS), as previously described (Ceyssens et al., 2008; Holtappels et al., 2015).

### Genome Accession Numbers

*Lactobacillus brevis* 100D8: CP015338, *L. brevis* ATCC 367: CP000416, *L. brevis* BDGP6: CP024635, *L. brevis* KB290: AP012167, *L. brevis* NCTC13768: LS483405, *L. brevis* NPS-QW-145: CP015398, *L. brevis* SA-C12: CP031185, *L. brevis* SRCM101106: CP021674, *L. brevis* SRCM101174: CP021479, *L. brevis* TMW 1.2108: CP019734, *L. brevis* TMW 1.2111: CP019743, *L. brevis* TMW 1.2112: CP016797, *L. brevis* TMW 1.2113: CP019750, *L. brevis* UCCLB521: CP031208, *L. brevis* UCCLB556: CP031174, *L. brevis* UCCLB95: CP031182, *L. brevis* UCCLBBS124: CP031169, *L. brevis* UCCLBBS449: CP031198, *L. brevis* ZLB004: CP021456, *L. brevis* phage 3-521: MK504444, *L. brevis* phage 521B: MK504443, *L. brevis* phage 3-SAC12: MK504442, *L. brevis* phage SAC12B: MK504446, *L. brevis* phage ATCCB: MK504445, *L. brevis* phage SA-C12: KU052488, and *L. brevis* phage LBR48: GU967410.

## Results and Discussion

### Prophage Identification and Characterization

*Lactobacillus brevis* contamination of beer is a consistent threat for breweries as its survival and growth in beer cause spoilage thus leading to product withdrawal and economic loss. The food and beverage industries aim to apply more natural, environmentally friendly and safer food preservation methods. Phage bioremediation or sanitation may represent a potential method to prevent bacterial growth and spoilage. LAB strains are known to carry prophage regions which, upon induction, may cause phage particle release and bacterial cell death.

The genomes of nineteen completely sequenced *L. brevis* strains were screened for the presence of prophage-encoding regions using PHASTER (Table 1). Of the nineteen bacterial strains of *L. brevis*, twenty-seven intact prophage sequences were predicted ranging from one to four prophage regions per strain. Twenty-three partial (marked as questionable and incomplete according to PHASTER analysis (Zhou et al., 2011; Arndt et al., 2016)) prophage regions were also identified among these strains. Four *L. brevis* strains do not appear to harbor intact prophage regions in their sequences, yet are predicted to carry remnant prophage sequences. The high number of prophage regions (intact and partial) identified shows that prophages are a very common occurrence in *L. brevis* genomes. Predicted intact prophage regions were manually examined and extracted for further analysis (general genome features are detailed in Table 3). Among the fifteen *L. brevis* strains whose genomes contain predicted intact prophage regions, *L. brevis* BDGP6 presented the highest number with four such prophage regions ranging in size from 42 to 74 Kb (Table 3). Interestingly, *L. brevis* strains SRCM101106 and SRCM101174 harbor an identical prophage, designated TPSRCM101106-3 and TPSRCM101174-3, respectively (100% nucleotide similarity across the full length of their genomes), this can be explained by the similarity between the two *L. brevis* host strains which share 99.8% nt sequence identity across 94% of their genomes. Similarly, *L. brevis* TMW1.2108 harbors two prophage regions that are nearly identical to those present in the genome of TMW1.2111 (TPTMW1-4 and TPTMW1-6, and TPTMW1-5 and TPTMW1-7 bearing 99.99 and 100% nucleotide similarity, respectively) and in *L. brevis* TMW1.2112 and TMW1.2113 sharing a 99.99% nucleotide identical prophage region (TPTMW1-1 and TPTMW1-2, respectively). These results are perhaps unsurprising as these *L. brevis* strains are more than 99% identical in their genome sequences but mostly differ in their plasmid content (Feyereisen et al., 2019a). The similarities observed in prophage content for some *L. brevis* strains might also be due to the common environment from which these strains have been isolated, i.e., food (South Korea) for *L. brevis* SRCM101106 and SRCM101174 and beer (Germany) for *L. brevis* TMW1.2108, TMW1.2111, TMW1.2112, and TMW1.2113. Integration sites (attL and attR sites) of prophages were identified and are presented in Supplementary Table S1. Diversity is observed among these integration sites; however, closely related prophages share the same sites, such as TPSRCM101106-3 and TPSRCM101174-3 or TPBDGP6-1 and TPSAC12-2. Several integration site sequences seem to be shared by certain prophages such as 5′-aaatcctgtactctcctt-3′ which was identified in five genomes. The presence of such sites acts as indicators of potential phage integration and movement, which is important in the context of phage and host evolution, fitness and adaptability in its ecological niche.

**TABLE 3:** General genome features of *Lactobacillus brevis* intact prophage regions and virulent phages.

**Strain name**	**Phages**	**Genome size (bp)**	**ORFs No**	**GC (%)**
**Lysogen**	**Prophages**			

100D8^∗^	TP100D8	41,993	62	44.6
ATCC 367	TPATCC367	56,030	78	43.4
BDGP6^∗^	TPBDGP6-1	41,938	63	42.1
	TPBDGP6-2	74,412	100	43.6
	TPBDGP6-3	46,262	71	44.9
	TPBDGP6-4	44,732	59	40.8
KB290^∗^	TPKB290-1	43,639	64	44.5
	TPKB290-2	47,873	70	44.3
SA-C12	TPSAC12-1	57,452	83	43.5
	TPSAC12-2	38,492	50	43.4
SRCM101106^∗^	TPSRCM101106-1	48,237	71	43.8
	TPSRCM101106-2	69,528	92	44.2
	TPSRCM101106-3	49,505	74	43.7
SRCM101174^∗^	TPSRCM101174-1	39,697	47	42.2
	TPSRCM101174-2	70,358	97	44.3
	TPSRCM101174-3	49,505	74	43.7
TMW 1.2108^∗^	TPTMW1-4	49,253	74	43.8
	TPTMW1-5	40,616	60	41.5
TMW 1.2111^∗^	TPTMW1-6	49,251	77	43.8
	TPTMW1-7	40,616	61	41.5
TMW 1.2112^∗^	TPTMW1-1	51,644	72	43.6
TMW 1.2113^∗^	TPTMW1-2	51,643	74	43.6
	TPTMW1-3	51,532	76	43.5
UCCLB95	TPMB095	66,077	80	44.1
UCCLBBS124	TPMB124	46,131	73	43.7
UCCLBBS449	TPMB449	50,224	72	43.8
ZLB004^∗^	TPZLB004	68,360	92	43.3

**Host**	**Virulent phages**			

UCCLB521	3-521	140,816	155	36.9
UCCLB521	521B	136,442	188	32.3
SA-C12	SAC12B	136,608	191	32.4
SA-C12	3-SAC12	41,292	61	40.0
ATTC 367	ATCCB	80,538	96	30.8

*^∗^These strains were not tested for prophage induction.*

Temperate phages can be detrimental for the host if, for example, they switch to a lytic state but they can also be beneficial to the host by carrying genes that will help the strain survive in its environment. These genes may encode phage-resistance systems such as Abi system or Sie proteins as previously identified (Mahony et al., 2008). Abortive infection systems were shown to block phage multiplication leading to the release of few particles and the death of the infected cells allowing the survival of the bacterial overall population (Chopin et al., 2005). Superinfection proteins were identified in *Lactococcus lactis* strains where they were shown to prevent DNA injection of certain phages without affecting phage adsorption (Mahony et al., 2008). Of the 19 *L. brevis* strains studied, nine strains were predicted to carry potential prophage-encoded Sie systems. Meanwhile six were predicted to carry potential prophage-encoded Abi system such as observed in the prophages TPMB124 and TPSRCM101174-3 based on BlastN analysis. These two prophage regions harbor the same potential Abi system which show similarity to the AbiL system identified in *Lactococcus lactis* (Deng et al., 1999). The presence of these potential resistance systems is predicted to confer resistance to the host against phage infection thus increasing the overall host fitness. The absence of such systems in *L. brevis* UCCLB521 or SA-C12 may explain their higher sensitivity to lytic phage infection. Conversely, the presence of potential phage-resistance systems (i.e., Abi) in the prophage of the beer-spoiling *L. brevis* strain UCCLBBS124 could explain its resistance against lytic phage infection (Feyereisen et al., 2019b).

### Prophage Inductions

**FIGURE 1:**
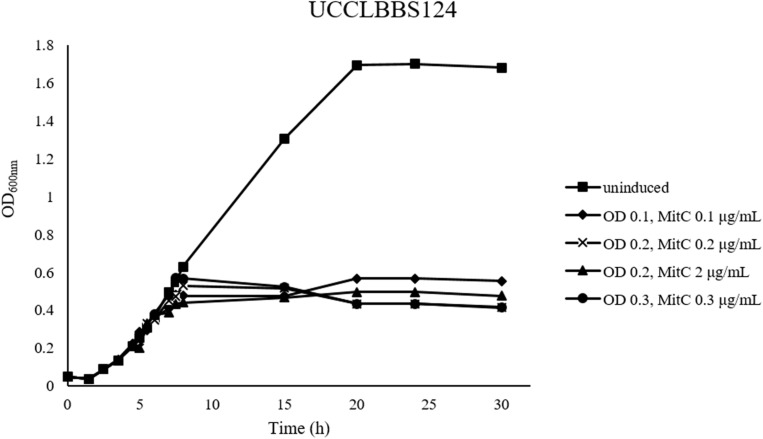
MitC induction profiles of *Lactobacillus brevis* UCCLBBS124. Different concentrations of the inducing agent MitC: 0.1, 0.2, 2, and 0.3 μg/mL were added to the culture after growth of the bacterial strain at an OD_600__nm_ of 0.1, 0.2, and 0.3, respectively. An uninduced culture was included as a control (indicated as “uninduced”).

Small scale prophage induction trials were performed for *L. brevis* strain UCCLBBS124. These trials were furthermore applied to ascertain the accuracy of the bioinformatic predictions of likely intact prophages within these genomes. MitC exposure was employed at sub-lethal (0.1–0.3 μg/mL) or lethal concentrations (2 μg/mL) to distinguish between genuine prophage induction-mediated cell lysis and cell death due to acute MitC toxicity. Prophage induction trials with *L. brevis* UCCLBBS124 generated different induction profiles (Figure 1), where both sub-lethal and lethal doses of MitC caused cell lysis, indicating that prophage induction may have occurred. Using the lowest concentration of MitC required for prophage induction in UCCLBBS124, phage inductions in other *L. brevis* strains were performed as described: cultures were grown and MitC was added at a sub-lethal concentration of 0.1 μg/mL MitC when the culture reached an OD_600__nm_ of 0.1. *L. brevis* strains ATCC 367, SA-C12, UCCLB95, UCCLBBS124, and UCCLBBS449 exhibited lysis upon addition of 0.1 μg/mL MitC indicating prophage induction, cell lysis and phage particle release.

Prophage inductions using 2% v/v formic acid or UV light exposure for 30 min were successful as indicated by PCR validation after phage DNA isolation. However, no apparent prophage induction was observed when acetic acid (2% v/v) or sodium hydroxide (2% v/v) was used as a potential inducing agent (data not shown). While in some cases formic acid and UV treatment appeared to cause cell death, phage particles were not observed by electron microscopy despite positive PCR assay results. This may be due to the detection limit of the microscopy approach limiting the ability to visualize the particles or to the poor growth characteristics of certain cultures (which appear to lyse) thus representing bacteriostasis rather than lysis.

### Validation of Prophage Induction

The five *L. brevis* strains that were available for testing showed lysis following induction using 0.1 μg/mL MitC. To further validate that the observed lysis corresponds to phage particle release, filtered cell free supernatants of the induced cultures were analyzed by (i) electron microscopy, (ii) DNA extraction to confirm the prophage sequence using a PCR-based technique, and (iii) structural proteome analysis using mass spectrometry. This was also performed to match a specific prophage sequence to virion morphology in cases where more than one intact prophage region was identified in a bacterial strain such as *L. brevis* SA-C12, which harbors two predicted prophage regions (Table 3).

**FIGURE 2:**
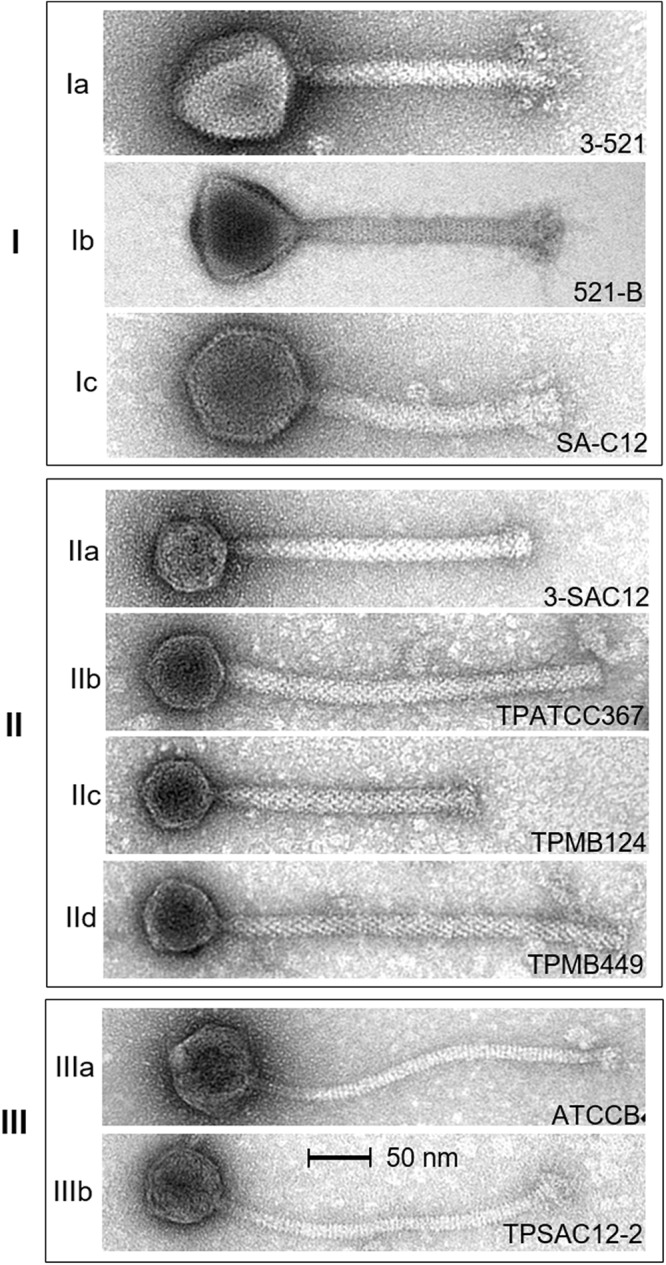
Electron micrographs of *L. brevis* phages representing morphotypes I to III. Ia, virulent phage 3-521; Ib, virulent phage 521B (Feyereisen et al., 2019b); and Ic, virulent phage SA-C12 (Deasy et al., 2011). IIa, virulent phage 3-SAC12 (Feyereisen et al., 2019b); IIb, temperate phage TPATCC367 induced from the *L. brevis* strain ATCC 367; IIc, temperate phage TPMB124 induced from the *L. brevis* strain UCCLBBS124; and IId, temperate phage TPMB449 induced from the *L. brevis* strain UCCLBBS449. IIIa, virulent phage ATCCB (Feyereisen et al., 2019b) and IIIb, temperate phage TPSAC12-2 induced from the *L. brevis* strain SA-C12. Temperate phages were induced from *L. brevis* strains using 0.1 μg/mL MitC.

Prophage induction attempts for five *L. brevis* strains, i.e., UCCLBBS124, ATCC 367, SA-C12, UCCLBBS449 and UCCLB95, resulted in the identification of intact virions which in one case was shown to bear morphological characteristics of *Siphoviridae* phages: TPSAC12-2 (induced from SA-C12) which is characterized by a long thin, non-contractile tail (Figure 2 and Table 3), while in three cases *Myoviridae* phages were obtained: TPMB124 (induced from UCCLBBS124), TPATCC367 (induced from ATCC 367), TPMB449 (induced from UCCLBBS449) characterized by a decorated contractile-tailed phage (Figure 2 and Table 3). No phage particles were visible following induction of strain UCCLB95, which indicates that the prophage may not be inducible (to produce detectable virions) under the tested conditions despite causing cell lysis highlighting the limitations of *in silico* analysis. Such predictions have previously been shown to require manual evaluation and assessment in lactococcal prophages thus necessitating induction trials employing various chemical agents and/or UV treatment (Kelleher et al., 2018).

**FIGURE 4:**
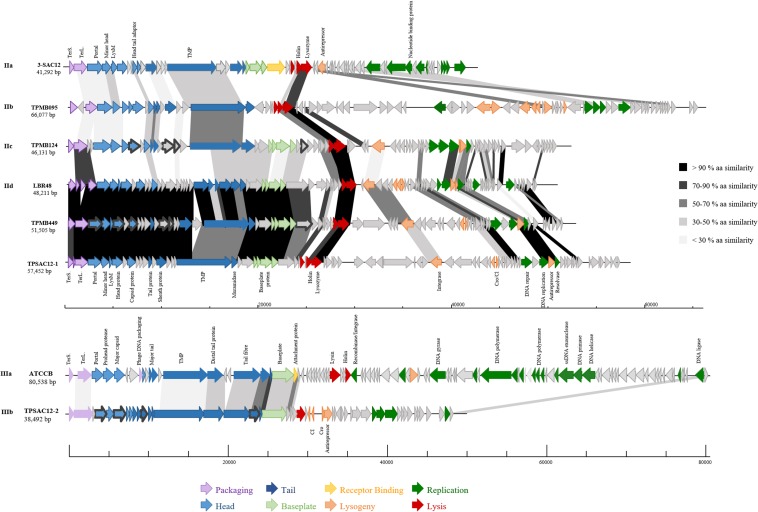
Genomic organization of representatives of *L. brevis* phages belonging to group II (*Myoviridae* Morphotype II phages): (IIa) 3-SAC12, (IIb) TPMB095, (IIc) TPMB124, (IId) LBR48, TPMB449 and TPSAC12-1, and group III: (IIIa) ATCCB and (IIIb) TPSAC12-2. The scale at the bottom of genomes is in base pairs. Each arrow represents an ORF, with the color representing the putative function of the encoded protein. Shaded boxes match the percentage of amino acid similarity between ORFs (TerS, small terminase; TerL, large terminase; and TMP, tape measure protein). Confirmed structural protein-encoding genes from mass spectrometry analysis are also highlighted (dark gray bold outline).

In parallel, PCRs targeting the gene encoding the Tape Measure Protein (TMP) of the temperate phages (Table 2) as well as phage structural proteome analysis using mass spectrometry (Table 4 and Figure 4) further validated the induction and prophage identification findings. Predicted proteins encoded within the morphogenesis module of the phage genomes of TPSAC12-2, TPMB124, TPATCC367, and TPMB449 were confirmed as structural proteins. Tail proteins, the major capsid protein and the portal protein were identified as structural proteins of the temperate phage TPSAC12-2 confirming the induction of this prophage from strain SA-C12 (no structural proteins matching those encoded by the prophage region TPSAC12-1 were identified, suggesting that this prophage was not induced upon MitC treatment) (Table 4). The minor capsid protein and three hypothetical proteins were confirmed as structural proteins of the prophage region TPMB124 (Table 4). More than ten proteins of TPATCC367 were identified as structural proteins among which the tape measure protein, the head protein and the major capsid protein (Table 4). Structural proteins of the temperate phage TPMB449 were identified by mass spectrometry including the capsid, head, and portal proteins. Some predicted structural proteins were not identified in the experimentally determined proteome, most likely due to their small size or their low relative abundance.

**TABLE 4:** Structural proteins extracted from purified phage particles by ESI-MS/MS.

**Phage**	**ORF**	**Putative function**	**No. of peptides**	**Sequence coverage (%)**
TPSAC12-2	SAC12_1335	Tail fiber protein	2	7.7
	SAC12_1361	Tail protein	5	30.2
	SAC12_1366	Major capsid protein	14	39.8
	SAC12_1368	Portal protein	4	19.8
TPMB124	UCCLBBS124_1395	Hypothetical protein	3	22.8
	UCCLBBS124_1396	Hypothetical protein	2	5.7
	UCCLBBS124_1402	Minor capsid protein	3	10.1
	UCCLBBS124_1457	Hypothetical protein	2	12.6
TPATCC367	LVIS_1073	Hypothetical protein	7	66.3
	LVIS_1080	Hypothetical protein	17	42.1
	LVIS_1081	Hypothetical protein	2	9.9
	LVIS_1084	Hypothetical protein	2	16.9
	LVIS_1085	Hypothetical protein	2	6.0
	LVIS_1087	Lysozyme	8	19.8
	LVIS_1088	Tape measure protein	24	13.2
	LVIS_1090	Hypothetical protein	6	38.9
	LVIS_1091	Hypothetical protein	7	49.4
	LVIS_1092	Hypothetical protein	12	56.4
	LVIS_1098	Major capsid protein	16	59.8
	LVIS_1099	Head protein	3	23.3
	LVIS_1102	Hypothetical protein	6	18.1
	LVIS_1128	Helicase	4	9.9
TPMB449	UCCLBBS449_1616	Hypothetical protein	5	10.6
	UCCLBBS449_1629	Hypothetical protein	3	33.3
	UCCLBBS449_1630	Structural protein	7	24.6
	UCCLBBS449_1636	Capsid protein	12	62.7
	UCCLBBS449_1638	Head protein	2	6.5
	UCCLBBS449_1641	Portal protein	3	6.2

*A minimum of two independent unique peptides or 5% coverage were used as threshold values.*

### Morphology of *L. brevis* Phages

Electron microscopic analysis of *L. brevis* phages available to date were gathered, providing insights into the morphological diversity among *L. brevis* phages. From these analyses, three distinct morphologies were observed (Figure 2). Firstly, *Myoviridae* phages which exhibit imposing head structures and contractile tails ranging from 166 to 201.9 nm incorporating an organelle at the tail tip called a baseplate were termed *Myoviridae* Morphotype I phages (Figure 2). They are represented by the virulent phages 3-521, 521B, SAC12B (Feyereisen et al., 2019b), and SA-C12 (Deasy et al., 2011).

Secondly, *L. brevis* phages such as 3-SAC12, TPATCC367, TPMB124 and TPMB449 also belong to the *Myoviridae* family, although their common morphology differs from that of the virulent *Myoviridae* Morphotype I phages mentioned above (Feyereisen et al., 2019b). In this case, their morphology is represented by a small head structure and a decorated tail with a discrete baseplate, termed here as *Myoviridae* Morphotype II phages (Figure 2). This morphology is similar to the one observed for the prophage LBR48 (Jang et al., 2011) and interestingly, varying tail lengths were observed for phages of this morphotype (Figure 2).

Finally, some *L. brevis* phages were classified as members of the *Siphoviridae* family. Phages from this group, ATCCB and TPSAC12-2, are characterized as possessing a long non-contractile tail and an icosahedral head and were termed Morphotype III phages (Figure 2).

### *L. brevis* Phage Phylogeny

**FIGURE 3:**
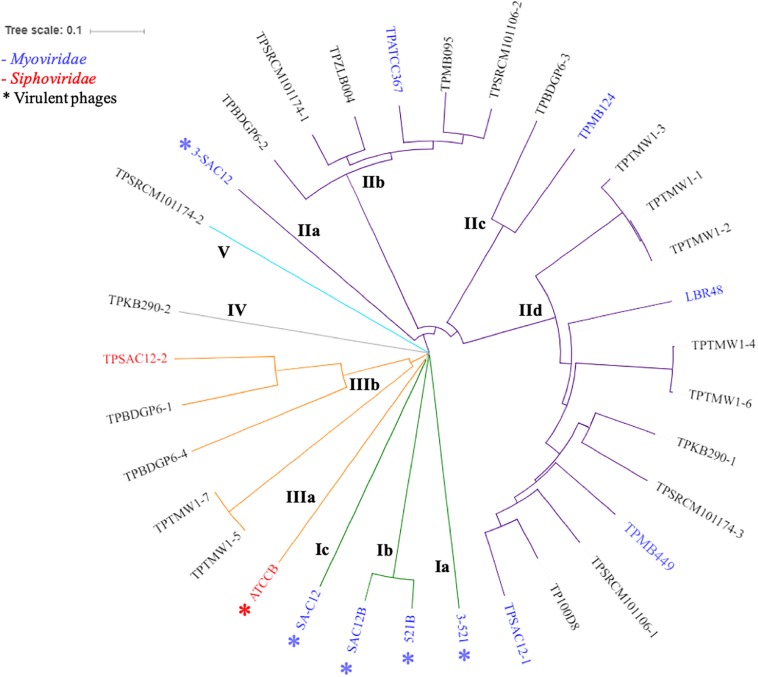
Proteomic tree of *L. brevis* phages available so far: virulent (annotated with ^∗^) and temperate phages. The phylogenetic analysis revealed five distinct groups (I to V) highlighted by different colors on the tree (green I, purple II, orange III, gray IV, and light blue V). *Myoviridae* phages are labeled in blue, while *Siphoviridae* phages are labeled in red.

In order to gain insight into the phylogeny of *L. brevis* prophages, a proteomic tree was constructed with all available sequences of *L. brevis* phages (virulent and temperate). Phylogenetic analysis resulted in the identification of five different groups (Figure 3) highlighting the apparent uniqueness of *L. brevis* phages. The virulent *Myoviridae* phages previously studied (Feyereisen et al., 2019b) were gathered in group I. Group II comprises twenty-one (one virulent and twenty temperate phages) *L. brevis* phages and phages of this group for which the family is known are all part of the *Myoviridae* family. Meanwhile group III gathers phages of the *Siphoviridae* family (when phage family is known). Groups IV and V each comprise of a single prophage sequence with distinct genetic composition and as yet unknown morphology (Figure 3). Interestingly, the majority of phages belong to the *Myoviridae* family, which is unusual among phages of LAB where *Siphoviridae* phages are more typically reported (Brüssow and Desiere, 2001; Casey et al., 2015; Kelleher et al., 2018).

### Classification of *L. brevis* Phages

To date, limited studies of *L. brevis* phages have been undertaken despite the commercial relevance of this bacterial species and its associated bacteriophages (Deasy et al., 2011; Jang et al., 2011). Here, we propose a classification of *L. brevis* phages similar to what has previously been undertaken for *Leuconostoc* phages (Kot et al., 2014) based on morphology, phylogeny and genomic diversity. The classification suggested here, divides *L. brevis* phages into five groups, I to V, linking the phylogeny and morphology analyses as described above.

The group I observed on the proteomic tree (Figure 3), gathers virulent *Myoviridae* Morphotype I phages (Figure 2). They are further divided into three subgroups based on their genetic diversity level, phage 3-521 (Ia), phages 521B and SAC12B (Ib) sharing 97% nucleotide similarity (88% coverage) and finally SA-C12 (Ic). The previously described (Feyereisen et al., 2019b) phages 3-521, 521B and SAC12B are characterized by a large genome size (>136 Kb), probably in line with their imposing head structure, and a high degree of synteny throughout their genomes. Phage SA-C12 presents a similar morphology, yet harbors a smaller genome (79 Kb) (Deasy et al., 2011). The genome of SA-C12 is quite divergent and seems to be missing a certain number of genes encoding for hypothetical proteins and proteins involved in the replication process compared to other phages of this group.

Group II (Figure 3) is represented by *Myoviridae* Morphotype II phages (Figure 2), thereby encompassing over half of the *L. brevis* phages. Genomic and morphological analysis of representative isolates suggest that all members of this group belong to the *Myoviridae* family. Based on their genetic diversity, group II phages are divided here into four subgroups (Figure 3). The subgroup IIa is comprised of the virulent phage 3-SAC12 (40 Kb) which has previously been described (Feyereisen et al., 2019b). Subgroup IIb is composed of six temperate phages, including TPATCC367 (Figure 2), which share around 90% nucleotide similarity (78% sequence coverage) and harbor a larger genome (average of 62 Kb) compared to other group II members. Subgroup IIc gathers temperate phages TPBDGP6-3 and TPMB124 with an average genome size of 46 Kb and sharing 92% nucleotide similarity (56% sequence coverage). The biggest subgroup, subgroup IId, comprises twelve temperate phages sharing at least 90% nucleotide similarity (55% sequence coverage) and harboring a genome with an average size of 49 Kb.

In depth comparative analysis highlighted the degree of amino acid similarity between these *Myoviridae* Morphotype II phages. Although divergence occurs within these phages, genomic synteny is observed and the genomes are organized into modules corresponding to DNA packaging, structure, lysis/lysogeny and replication (Figure 4). Different tail lengths were observed for phages belonging to the group II (Figure 2) and, as previously described, the length of the tail seems to be linked to the size of the TMP gene (Mahony et al., 2016a). Indeed, phages belonging to the group IIc and presenting the smallest tail (Figure 2), have on average a TMP-encoding gene of 4.82 Kb, while phages belonging to the group IIb and IId which present the longest tailed-phages (Figure 2) possess a TMP-encoding gene with an average size of 5.67 and 5.50 Kb, respectively (Figure 4). Proteins encoding holin and lysozyme (lysin) are highly conserved across the representative phages (more than 70% amino acid (aa) similarity). The virulent phage 3-SAC12 (IIa) and the temperate phages TPMB095 (IIb) and TPMB124 (IIc) revealed low levels of similarity (between 30 and 50% aa similarity) across their structural modules. Surprisingly, only temperate phage TPMB095 lacks sequence homology with other phages of this group in the region encoding the baseplate structure. The absence of such genes may be partly responsible for the inability of TPMB095 to form functional phage and thus was not inducible. Furthermore, the genome appears quite decayed with several transposase elements interjecting the genome and the overall genome appears to lack architectural conservation compared to many other phages of LAB. *L. brevis* prophages LBR48, TPMB449, and TPSAC12-1 (IId) share a high level of similarity (more than 70% aa similarity) across the entire packaging, structural and lysis module (Figure 4). They also share between 30 and 50% aa similarity between their predicted integrase proteins.

Group III (Figure 3) is represented by temperate and virulent phages, and gathers members of the *Siphoviridae* family (e.g., ATCCB and TPSAC12-2) characterized as Morphotype III (Figure 2). Other lysogenic bacteria carrying prophages of this group were not available for prophage induction, therefore their morphotype remains unknown. It is likely that they belong to the *Siphoviridae* family as they are most closely related to the siphophage TPSAC12-2. The group III is divided into two subgroups separating the virulent phage ATCCB (IIIa) with a genome size of approximately 80 Kb and five temperate phages (IIIb) harboring smaller genomes (size of around 40 Kb) and among which the siphophage TPSAC12-2 can be found (Figure 3). Representatives of group III for which electron microscopy images (where available) were chosen for further comparative analysis (Figure 4). Virulent phage ATCC-B and temperate phage TPSAC12-2 share synteny in terms of genome organization with the DNA packaging module followed by the structural module, the lysis/lysogeny module and the replication module. The two phages share a low level of similarity, yet synteny and aa similarity of around 30% across the DNA packaging and structural modules with proteins encoding terminase, capsid, and tail morphogenesis-associated functions were observed. Temperate phage TPSAC12-2, unlike virulent phage ATCC-B, harbors ORFs encoding predicted lysogeny functions, such as a Cro/Cl repressor and an antirepressor (Figure 4).

Groups IV and V contain single members, i.e., prophages TPKB290-2 and TPSRCM101174-2, respectively, and do not share any significant sequence similarity to the other groups. The morphology of these phages is unknown and based on their genome analysis it is difficult to derive assumptions on the morphology/classification of TPSRCM101174-2. However, based on TPKB290-2 sequence data, a gene encoding a sheath protein is predicted suggesting that this phage is a *Myoviridae* member.

Induced temperate phages were tested for their potential ability to infect the seven *L. brevis* strains that were available in our collection (Table 1). However, these phages did not show any activity against the tested strains. Interestingly, the repressors encoded by prophages of *L. brevis* do not share widespread sequence homology indicating that homo-immunity based on the activity of repressors is not the basis of the observed phage-resistance. It is likely that the observed phage-resistance is due to the use of alternative receptors on the cell surface by different phage groups or through the activity of phage-resistance mechanisms such as abortive infection systems.

In order to evaluate the diversity of *L. brevis* phages in relation to other *Lactobacillus* phages, a proteomic tree was created gathering *L. brevis* phages described in this study as well as previously sequenced *Lactobacillus* phages (Supplementary Figure S1). The phylogenetic tree clearly shows the distinct grouping of *L. brevis* phages separately to other *Lactobacillus* phages. However, some *L. brevis* phages showed similarity with phages infecting other species such as observed for the *L. brevis* phage SA-C12 and the *Lb. plantarum* phage 8014-B2.

## Conclusion

The genomes of nineteen bacterial strains of *L. brevis* analyzed in this study all harbor predicted prophage regions and twenty-seven intact prophage regions were identified. Only four *L. brevis* strains do not appear to contain intact prophage regions in their genomes. These numbers reveal the high incidence of prophages among *L. brevis* genomes with an average of 1.4 prophage region per strain. Of the five *L. brevis* strains available for prophage induction trials, four prophages were successfully induced and morphologically characterized by electron microscopy indicating a significant incidence of inducibility of these temperate phages. Electron microscopy observations, genome sequence analyses, and phylogeny allowed the classification of *L. brevis* phages into five groups, I to V. The results show substantial diversity among *L. brevis* phages and interestingly these entities are mostly represented by members of the *Myoviridae* family, unlike the majority of LAB phages.

The potential of prophages as antimicrobial agents in beer fermentation is a promising alternative to currently employed processes. Prophage induction in beer-spoiling *L. brevis* strain could be used during the cleaning process (such as coupled with sanitizers and/or UV treatment). However, this approach presents challenges and hurdles (i.e., scale-up for industrial settings and phage-encoded resistance mechanisms) that need to be addressed before their use in industry as bacterial spoilage control. To date, very few *L. brevis* phages have been characterized and the identification and characterization of additional phages will provide greater insights into *L. brevis* phage biodiversity and their potential application and role in food spoilage prevention.

## Data Availability Statement

All datasets generated for this study are included in the manuscript/Supplementary Files.

## Author Contributions

MF performed the experiments, annotation, genomic, and proteomic analysis. HN and CF carried out the electron microscopy analysis. J-PN carried out the mass spectrometry analysis. DS, JM, TO’S, and VB provided the materials and strains. MF, JM, TO’S, and DS were involved in project design and wrote the manuscript. All authors read and approved the final manuscript.

## Conflict of Interest

VB and TO’S are employees of Heineken. The remaining authors declare that the research was conducted in the absence of any commercial or financial relationships that could be construed as a potential conflict of interest.
