# Supplementary material for: Biodiversity and Classification of Phages Infecting Lactobacillus brevis
Source: Front Microbiol. 2019 Oct 16;10:2396. doi: 10.3389/fmicb.2019.02396 (PMC6805780; doi:10.3389/fmicb.2019.02396)
Supplement: Supplementary file 2 [file Data_Sheet_2.docx]

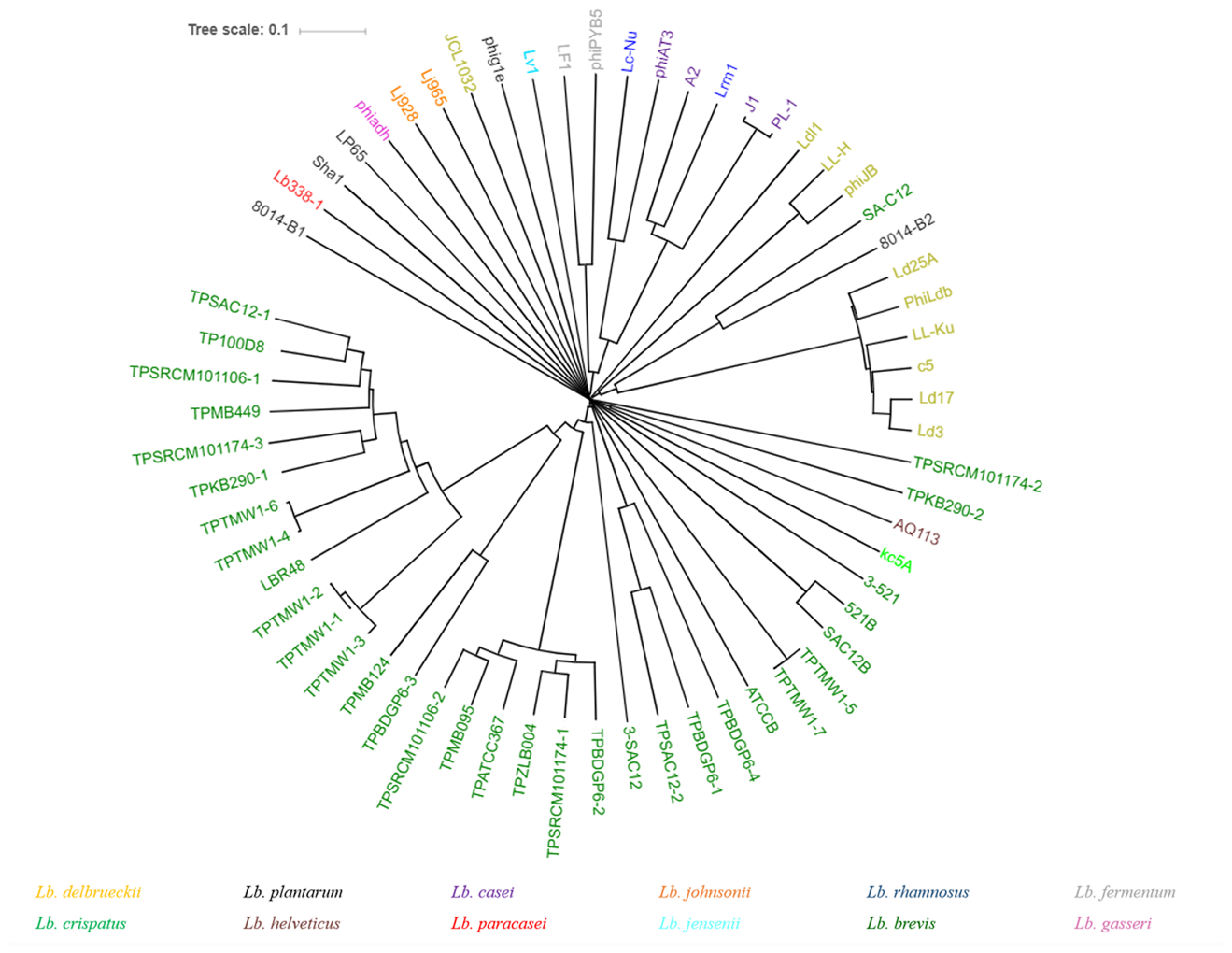


**Supplementary Figure 1.** Proteomic tree of *Lb. brevis* phages characterized in this study and all *Lactobacillus* phages sequenced to date. Color coding indicates the host species for each phage.
